# Physiological and anatomical aspects of the reproduction of mice with reduced Syndecan-1 expression

**DOI:** 10.1186/s12958-019-0470-2

**Published:** 2019-03-02

**Authors:** Christina Gougoula, Alexandra P. Bielfeld, Sarah J. Pour, Jan-S. Krüssel, Martin Götte, W. Peter M. Benten, Dunja M. Baston-Büst

**Affiliations:** 1 Central Unit for Animal Research and Animal Welfare Affairs (ZETT) of the Heinrich-Heine-University of Düsseldorf, Universitätsstraße 1, 40225 Düsseldorf, Germany; 2 Department of OB/GYN and REI (UniKiD), University Hospital Düsseldorf, Moorenstraße 5, 40225 Düsseldorf, Germany; 3 Department of Gynecology and Obstetrics, Münster University Hospital, Albert-Schweitzer-Campus 1, 48149 Münster, Germany

**Keywords:** Proteoglycan, Embryo implantation, Sperm, Development, Cycle, Syndecans

## Abstract

**Background:**

Syndecan-1 is a heparan sulfate proteoglycan acting as a co-receptor for cytokines and growth factors mediating developmental, immunological and angiogenic processes. In human, the uteroplacental localization of Syndecan-1 and its reduced expression in pregnancy-associated pathologies, such as the intrauterine growth restriction, suggests an influence of Syndecan-1 in embryo-maternal interactions. The aim of the present study was to identify the effect of a reduced expression of Syndecan-1 on the reproductive phenotype of mice and their progenies.

**Methods:**

Reproductive characteristics have been investigated using animals with reduced Syndecan-1 and their wildtype controls after normal mating and after vice versa embryo transfers. Female mice were used to measure the estrus cycle length and the weight gain during pregnancy, as well as for histological examination of ovaries. Male mice were examined for the concentration, motility, viability and morphology of spermatozoa. Organs like heart, lung, liver, kidney, spleen, brain and ovaries or testes and epididymis of 6-month-old animals were isolated and weighed. Statistical analyses were performed using two-tailed students t-test with *P* < .05 and *P* < .02, chi square test (*P* < .05) and Fisher’s Exact Test (*P* < .05). A linear and a non-linear mixed-effects model were generated to analyze the weight gain of pregnant females and of the progenies.

**Results:**

Focusing on the pregnancy outcome, the Syndecan-1 reduced females gave birth to larger litters. However, regarding the survival of the offspring, a higher percentage of pups with less Syndecan-1 died during the first postnatal days. Even though the ovaries and the testes of Syndecan-1 reduced mice showed no histological differences and the ovaries showed a similar number of primary and secondary follicles and *corpora lutea*, the spermatozoa of Syndecan-1 reduced males showed more tail and midpiece deficiencies. Concerning the postnatal and juvenile development the pups with reduced Syndecan-1 expression remained lighter and smaller regardless whether carried by mothers with reduced Syndecan-1 or wildtype foster mothers. With respect to anatomical differences kidneys of both genders as well as testes and epididymis of male mice with reduced syndecan-1 expression weighed less compared to controls.

**Conclusions:**

These data reveal that the effects of Syndecan-1 reduction are rather genotype- than parental-dependent.

## Background

Heparan sulfate (HS) proteoglycans (PGs) are ubiquitous frequent glycoproteins with one or more HS chain/s that can bind cytokines and growth factors and hence generate gradients influencing developmental, immunological and angiogenic processes [1]. Syndecans (SDCs) belong to the well-studied family of HSPGs which consists of 4 genes (*Sdc1 to 4*) [1]. So far, *Sdc1*^*−/−*^ knock-out (KO) mouse models revealed the participation of SDC1 in cancer cell proliferation and apoptosis [2, 3], as well as in angiogenesis [4].

The present study focuses on the reproductive phenotype of heterozygous *Sdc1*^*+/−*^ mice, as studies from our group previously showed the involvement of SDC1 at the embryo-maternal interface in vitro regulating the secretion of chemokines and angiogenic factors during decidualization, implantation and implantation-associated apoptosis in human endometrial epithelial and stromal cells [5–7]. SDC1 has been shown to be expressed in the human endometrium throughout the menstrual cycle [8] and could be associated with numerous human pregnancy pathologies based upon an insufficient implantation process. The reduced placental expression of SDC1 could be correlated with intrauterine growth restriction [9], preeclampsia [10], and hemolysis, elevated liver enzymes and low platelet count (HELLP) syndrome [11], whereas elevated placental SDC1 expression reduced the risk for preterm birth [12].

Even though the *Sdc1* mouse model is widely used in animal research, the reproductive phenotype has not been investigated, yet. In general, the characteristics of the remarkably short reproductive period and parturition interval render the mouse a valuable tool for studying the reproductive phenotype [13]. Mice have a short window for embryo implantation [14, 15], that lasts less than 24 h, a time frame that reduces the chances of a successful implantation in case of targeted mating. Therefore, many studies tried to establish an identification system for the estrous cycle phases [16] until Stockard and Papanicolaou developed a histological examination focusing on vaginal cells [17] including epithelial cells, cornified cells and leukocytes [18, 19].

The aim of the present study was to examine the reproductive phenotype of the *Sdc1*^*+/−*^ mouse, since for practical and ethical reasons the in vivo examination in human is not possible during an ongoing pregnancy. We focused on heterozygous *Sdc1*^*+/−*^ mice with a reduced concentration of SDC1 instead of *Sdc1*^*−/−*^ mice because a downregulation may reflect a possible dysregulation in human more closely rather than a complete absence of SDC1, which can be expected to be a rare event. Concentrating on reproductive characteristics, the ovaries, testes and germline cells were examined followed by pregnancy characteristics after normal mating and after vice versa embryo transfers. Consecutively the offspring with respect to viability and weight gain from birth to adolescence have been studied because a potential slow postnatal growth due to a possibly reduced lactation was of interest, as it has been described in the literature, that animals with a complete knock out of SDC1 present an impaired mammary ductal development [3]. Therefore, the individual reproductive characteristics of the *Sdc1*^*+/−*^ mouse compared to WT mouse were investigated to reveal if the origin of the SDC1 effect is of embryonic, maternal and/or paternal source.

## Methods

### Animals

Planning and conduction of the experimental procedures as well as maintenance of the animals was carried out in accordance to the German Guide for the Care and Use of Laboratory animals after they were approved by the State Office for Nature, Environment and Consumer Protection (LANUV, State of North Rhine-Westphalia, Germany). Mice were maintained at 20–24 °C on a 12 h light/12 h dark cycle with food (ssniff Spezialdiäten GmbH, Soest, Germany) and water ad libitum. *Sdc1* KO (*Sdc1*^*−/−*^) mice were originally generated on a C57BL/6J background, C57BL/6J.129Sv-*Sdc1*^*tm12MB*^ [20] by completely backcrossing for 10 generations.

### Quantification of SDC1 expression

Tail biopsies were genotyped according to the FELASA guidelines [21]. For the quantitative measurement of SDC1 the mouse SDC1 ELISA Kit (biorbyt, San Francisco, California, USA) was applied. Tail biopsies from 15 *Sdc1*^*−/−*^, 17 *Sdc1*^*+/−*^ and 50 WT mice were homogenized and lysed in tissue lysis buffer (0.5% (*v*/v) octylphenoxypolyethoxyethanol, 0.5% (*w*/*v*) sodium deoxycholate, 0.1% (w/v) sodium dodecyl sulfate, 50 mM Tris-HCl (pH 7.5), 150 mM NaCl, 1% (v/v) protease inhibitor cocktail (Sigma-Aldrich, Munich, Germany) and 100 μl of the homogenate was used to perform the ELISA according to the manufacturer’s instructions. Furthermore, 1 μl of the homogenate was used for whole protein quantification via BCA protein assay (Thermo Scientific, Waltham, Massachusetts, USA) to normalize the amount of SDC1.

### Detection of estrous cycle and breeding characteristics

Vaginal smears from 8-weeks-old females of both *Sdc1*^*+/−*^ (*n* = 29) and WT (*n* = 34) groups were extracted daily for 12 days at the same time [22] and observed under the microscope (Carl Zeiss Fixed Stage Standard Microscope, 10x Objective, Oberkochen, Germany). The proportion of nucleated epithelial cells, cornified squamous epithelial cells and leukocytes was counted [22].

The duration of pregnancy and the weight gain during pregnancy were constantly studied with a particular number of females: 6 *Sdc1*^*+/−*^ females and 4 controls in single matings and 5 *Sdc1*^*+/−*^ and 5 WT females which were mated individually and continuously for a period of 4 months. The weight (Dipse digital scale TP500, Oldenburg, Germany) of the pregnant *Sdc1*^*+/−*^ and control females was monitored the day before mating, indicated as the day before the presence of a vaginal plug (day 0), as well as on day 4, 8, 12, 16, 18 after mating and then every day until birth.

### Organ isolation

The progeny of both groups was weighed directly after birth, then every 3 days until the 60th day and subsequently once in 10 days until day 200. The following organs of 200-days-old male and female *Sdc1*^*+/−*^ and WT mice have been weighed: heart, lung, liver, kidney, spleen and brain (Mettler Toledo AE50, Dorsten, Germany). For the selective examination of implantation sites, uteri from 8-week-old females (*Sdc1*^*+/−*^ and WT, each 30 animals) at embryonic day 6 of pregnancy were extracted. Additionally, ovaries were isolated and fixed in formalin for further histological hematoxylin and eosin examination [23]. Three investigators assessed the number and morphology of the primary and secondary/tertiary follicles. Both testes and epididymis of 6-month-old males were assessed for sperm analysis (*Sdc1*^*+/−*^/WT males: *n* = 28/24). The caput and corpus epididymis were weighed together, the cauda alone. Paired organs were weighed separately and the mean value was calculated. Additional animals were used for the weighing of adults organs apart from the ones that were weighed up to day 200 so those in totals a minimum of 49 animals were examined.

From the vice versa embryo transfers (see below) the organs of 8 *Sdc1*^*+/−*^ males, 6 *Sdc1*^*+/−*^ females, 3 WT males and 5 WT females were also isolated and weighed (Mettler Toledo AE50, Dorsten, Germany).

Organ to body weight ratios were calculated and were considered more useful because of the body weight differences [24–26].

### Embryo transfer

Female mice were intraperitoneally superovulated using 5 IU PMSG (Intergonan® 240 IE/ml, MSD Tiergesundheit, Unterschleißheim, Germany) and 5 IU hCG (Predalon® 5000 IE, Essex Pharma GmbH, Waltrop, Germany) 48 h later, followed by mating [27]. On day 1.5 after HCG administration, egg donors were sacrificed, their oviducts extracted and the embryos at the 2-cell stage flushed using M2 medium (Sigma-Aldrich, Munich, Germany). An average number of 12 2-cell embryos were transferred in the oviduct of pseudopregnant recipient foster mothers [27] of the opposite mouse line (*Sdc1*^*+/−*^ embryos into 4 WT and WT embryos into 3 *Sdc1*^*+/−*^ recipients). Pups from these vice versa embryo transfers were monitored as mentioned above until day 200 (*Sdc1*^*+/−*^ males: *n* = 8, *Sdc1*^*+/−*^ females: *n* = 6; WT males: *n* = 3, WT females: *n* = 5).

### Male reproductive characteristics

Adult non-breeder males (*Sdc1*^*+/−*^: *n* = 28; WT: *n* = 24) were euthanized, the anogenital distances measured [28], and the cauda, corpus, caput epididymis and testes isolated and weighed. The testes and the caput-corpus epididymis were fixed in Bouin’s solution (RAL Diagnostics, Martillac, France) for immunohistochemical analysis [23], whereas the cauda epididymis were placed into 2 ml hypertonic saline buffer [29] in a 35 mm culture dish. The epididymis were minced and the sperm were allowed to swim out of the tissue by incubating the dish in a 37 °C incubator (MCO-5 AC, Sanyo, Eschborn, Germany). After 30 min the suspension was centrifuged (Universal 320R centrifuge, Hettich, Vlotho, Germany) for 5 min at 0.1 rcf (relative centrifugal force) and the precipitate used for further analysis. Two independent investigators assessed the histology of the testes and the sperm concentration, viability and morphology by microscopical examination. The number of motile and immotile sperm cells was counted twice using a disposable Makler counting chamber (CV 1010–102, Cell Vision, Heerhugowaard, The Netherlands) under a light microscope (Carl Zeiss Fixed Stage Standard Microscope, 10x Carl Zeiss Objective, Oberkochen, Germany).

Regarding sperm viability, the number of viable and nonviable spermatozoa was counted after staining in 0.5% eosin solution twice in a Neubauer counting chamber (Fast Read 102®, Biosigma S.r.l., Cona, Italy) under a light microscope (Carl Zeiss Fixed Stage Standard Microscope, 40x Carl Zeiss Objective). Sperm morphology was determined after staining using the SpermacStain® kit (FertiPro N.V., Beernem, Belgium) according to manufacturer’s instructions and the literature [30]. The percentage of normal, head-, acrosome- and tail-defective spermatozoa in a total of 100 cells was calculated twice for the air-dried smears under a Carl Zeiss Fixed Stage Standard Microscope by two independent investigators (Neofluar 100x Carl Zeiss Oil Objective).

### Statistical analysis

Statistical analysis was performed using two-tailed student’s t-test (*P* < 0.05) for the number of implantation sites, born and dead pups, litter sizes, organ weights and anogenital distances. The two-tailed t-test with Bonferroni adjustment (*P* < 0.02) was applied to compare the SDC1 amount in *Sdc1*^*−/−*^, *Sdc1*^*+/−*^ and WT animals, the weight of the mice at day 0, 33 and 60 of their development, chi square test (*P* < 0.05) for sperm analysis and Fisher’s Exact Test (*P* < 0.05) for the mouse cycle data. Results are depicted as mean ± S.E.M. A linear mixed-effects model was generated to analyze the weight gain of pregnant females (R statistical package, Version 3.3.2.). Included predictors were observation days, mouse line (*Sdc1*^*+/−*^, WT) and the interaction between time and mouse line (*P* < 0.05). The correlation coefficient Spearman’s Rho (ρ) was employed for weight gain depending on litter size. Concerning the weight measurements of the progeny from day 0 to 200 a nonlinear mixed-effects model (weighing curves, R statistical package, Version 3.2.4, lme4 packet for linear mixed-effects models, Lattice packet for the graphics) [31] with the form *y* = *α* − *β* ∗ *γ*^*x*^ was applied. The fixed effects are the group effects (*Sdc1*^*+/−*^, WT, mother/foster) for each parameter α, ß and γ of the nonlinear curve. Random effect components were defined as the deviations of individual parameters with respect to the average of the corresponding group. The y-value represents mouse weight at a certain time point x-value in the development of the mouse. α indicates the maximum possible weight, ß the difference between the maximum and starting weight. γ is growth rate specific for each animal or group. Thus, the growth development of the *Sdc1*^*+/−*^ and WT mice is calculated from the maximum weight α and the growth rate ßγ^x^ according to the formula given. The level of significance for each variable is given at each table in the results part and the combination of the 3 variables gives the overall level of significance (*P* < 0.05).

## Results

### Proof of the SDC1 reduction

**Fig. 1:**
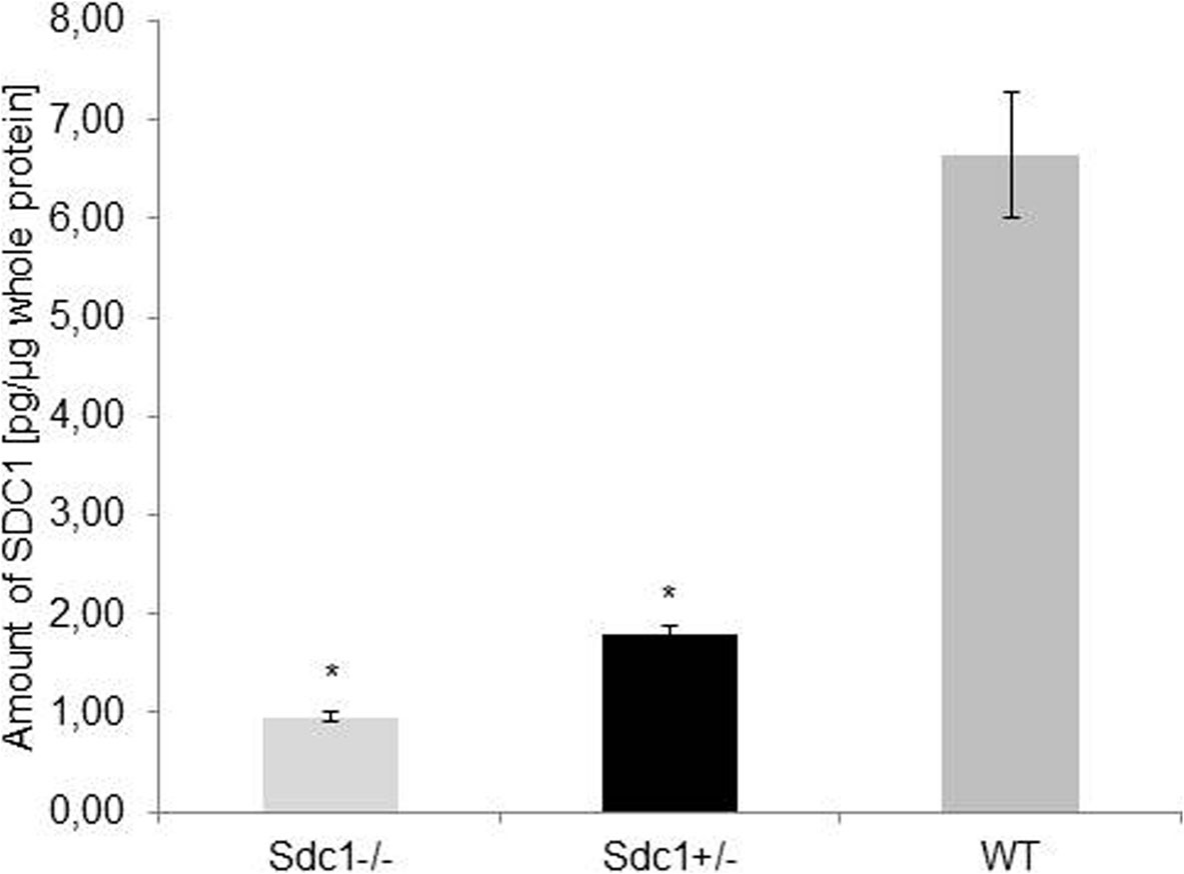
Quantification of SDC1. Measurement of the SDC1 in tail biopsies of *Sdc1*^*−/−*^ (light grey bar; *n* = 15), *Sdc1*^*+/−*^ (black bar; *n* = 17) and WT (dark grey bar; *n* = 50) mice using the ELISA method. Data were normalized to the total amount of protein (*P* < 0.02; two-tailed t-test with Bonferroni adjustment)

Quantitative measurement of SDC1 revealed that *Sdc1*^*+/−*^ mice had 60% less amount of protein in comparison to the WT mice (Fig. 1, *P* < 0.01). This difference was independent from gender and age.

### Mouse cycle

Physiologically, the estrous stages are: pro- (P), estrus (E), met- (M) and diestrus (D). The first cycle for each female started with the actual cycle day of sampling and was completed with M or D after an E.

**Fig. 2:**
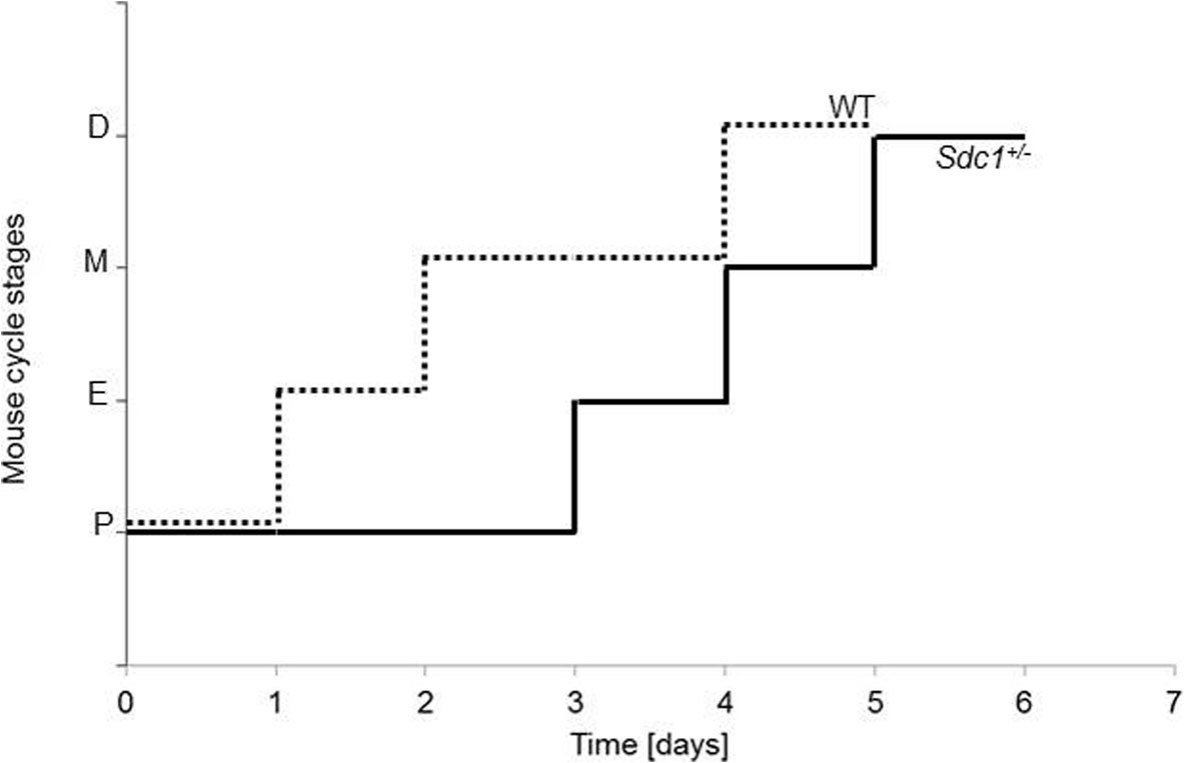
Representative estrous cycle of a *Sdc1*^*+/−*^ and a WT female. Cycle stages are shown as a line graph with estrous cycle (E, P, M, D) on the y axis and the duration (days) on the x axis

Sexual mature females of the *Sdc1*^*+/−*^ and WT group had an average number of 1.79 ± 0.11 and 1.91 ± 0.09 cycles respectively. Eight *Sdc1*^*+/−*^ and 6 WT females underwent only 1 cycle, 18 *Sdc1*^*+/−*^ and 24 WT females showed 2 cycles and 2 *Sdc1*^*+/−*^ and 3 WT had 3 cycles. In Table 1 an overview of the sequential arrangement of each stage per cycle is depicted (1–6 days). For the *Sdc1*^*+/−*^ and WT group, the average cycle duration was 5.02 ± 0.19 and 4.59 ± 0.15 days respectively 48% of the *Sdc1*^*+/−*^ and 40% of the WT mice underwent a 4-day cycle, 16% of *Sdc1*^*+/−*^ and 37% of WT a 5-day (*P* < 0.05) and finally, 24% of *Sdc1*^*+/−*^ and 3% of WT females had a cycle of 6 days (*P* < 0.05). A representative cycle of a *Sdc1*^*+/−*^ and a WT female is depicted in Fig. 2.

**Table 1:** Number of individual episodes of Proestrus (P), Estrus (E), Metestrus (M) and Diestrus (D)

Stage	P				E			M				D			
Days	1	2	3	4	1	2	3	1	2	3	4	1	2	4	6
*Sdc1* ^*+/−*^	11	32	6	1	45	4	1	33	14	2	0	20	2	0	1
WT	38	18	4	1	51	11	1	40	19	1	1	28	0	1	0

Concerning the observed irregular cycles (6 for the *Sdc1*^*+/−*^ and 5 for the WT group), 3 *Sdc1*^*+/−*^ females showed 3 cycles in absence of E, 2 cycles without P and only 1 that showed no M. On the contrary, for the WT females there was only 1 female with no E stage and all other 4 showed unterminated E cycles with no M and/or D stage after only 1 or more days of E.

### Characteristics of the female reproductive phenotype and the progeny

30 females of each group showed a vaginal plug after mating and 53% of the *Sdc1*^*+/−*^ and 47% of the WT females showed implantation sites on embryonic day 6 with an average number of 8.00 ± 0.45 for the *Sdc1*^*+/−*^ and 7.29 ± 0.53 for WT. The histological examination of the ovaries revealed no significant differences for the number of either primary, secondary or tertiary follicles or *corpora lutea* (data not shown).

The duration of pregnancy for the *Sdc1*^*+/−*^ and the WT females in the breeding setting was for the *Sdc1*^*+/−*^ 20.68 ± 0.47 and WT 20.89 ± 0.56 days with a range of 18 to 26 days. The statistically different mean initial weight (day 0) of *Sdc1*^*+/−*^ and WT females was 24.37 ± 0.83 g vs. 26.95 ± 0.98 g respectively (*P* < 0.05). During the course of pregnancy the *Sdc1*^*+/−*^ females gained 15.05 ± 0.53 g on average and gave birth to 7.36 ± 0.40 pups. The minimum weight gain was 9.65 g with a litter size of 5 and the maximum was 21.10 g with 10 pups born. The WT females gained 16.37 ± 0.88 g on average during pregnancy and gave birth to 6.37 ± 0.58 pups. The minimum weight gain was 8.70 g (3 pups) and the maximum 23.35 g (10 pups).

**Fig. 3:**
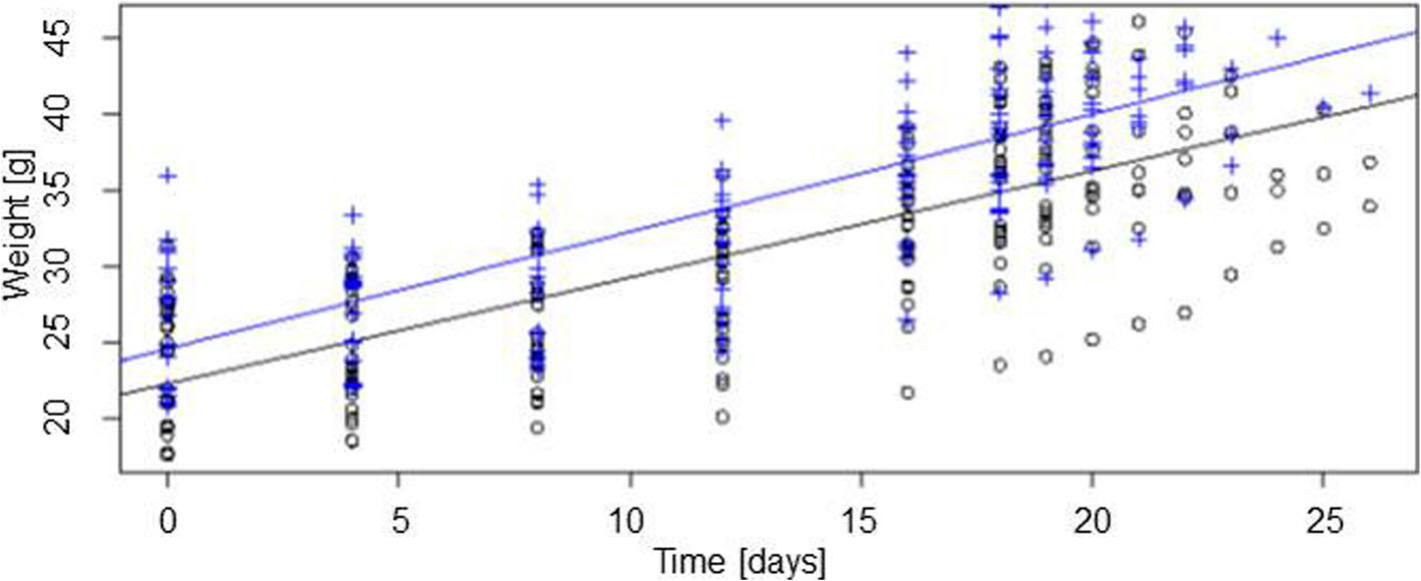
Weight gain of pregnant females. Increase in weight of *Sdc1*^*+/−*^ (black circles; *n* = 11) and WT (blue crosses; *n* = 9) females during the course of pregnancy calculated by a linear mixed-effects model

Regarding the course of pregnancy the WT females were heavier than the *Sdc1*^*+/−*^ females with a comparable weight gain per day (Fig. 3).

In case of consecutive litters, a moderate Spearman’s Rho correlation coefficiency (*ρ* = 0.53) between the litter size and the weight gain was found for the *Sdc1*^*+/−*^ group and a very strong association for the controls (*ρ* = 0.81).

**Fig. 4:**
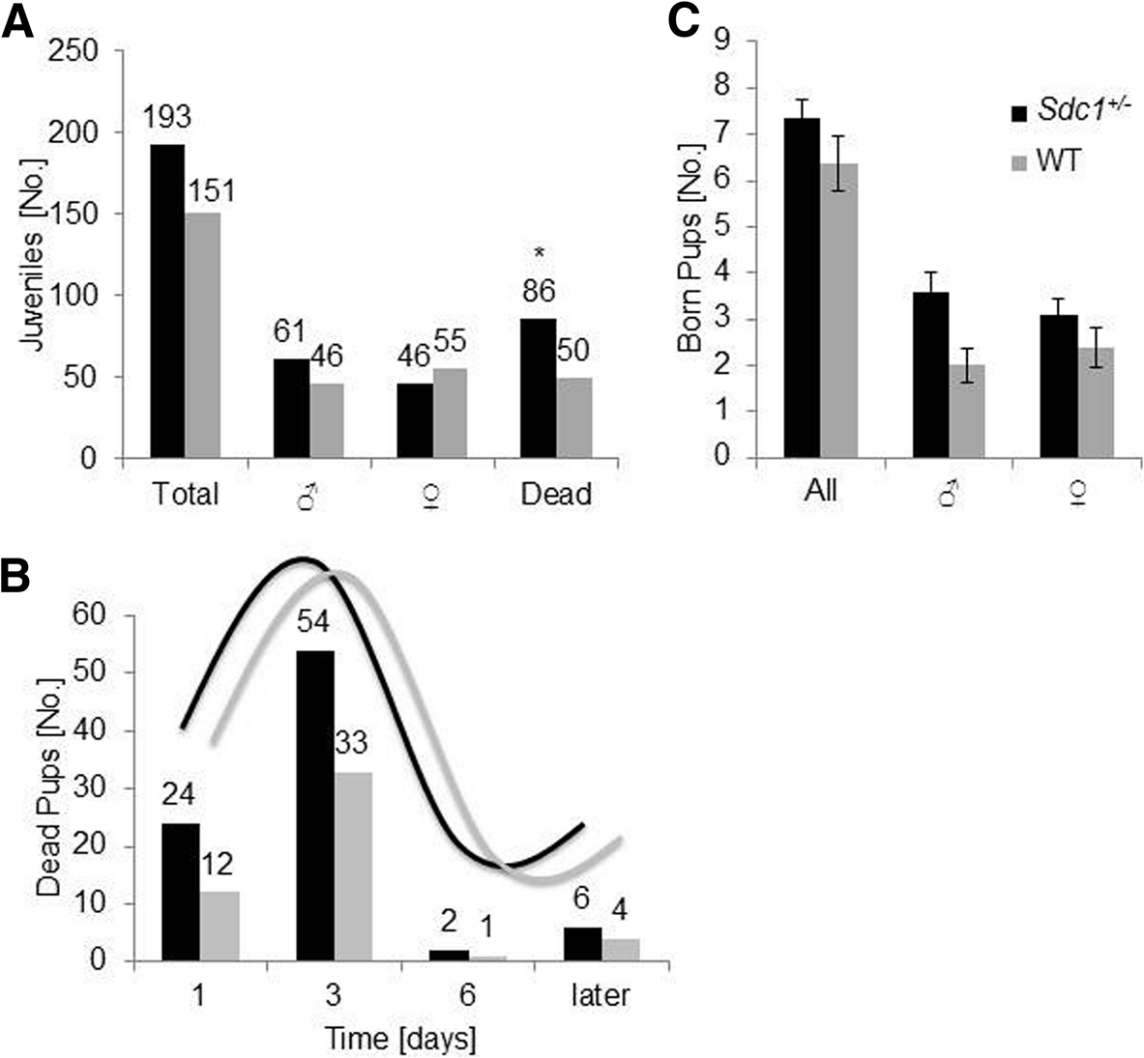
Observation of pregnancy outcome after mating. **a** Total number (25 SDC-reduced litters, 193 born pups, 23 WT litters: 151 born pups) of juveniles before and after gender determination and weaning (statistical significance between the numbers of dead juveniles of the two groups is indicated with an asterisk (*P* < 0.05; two-tailed t-test)). **b** Subdivision according to the day of death. The curves above the columns describe the sinusoidal death pace from day one to 6 and later. **c** Mean litter size before and after gender determination excluding the dead pups

Focusing on the development of the progenies, a total of 193 *Sdc1*^*+/−*^ pups (25 litters) and 151 WT pups (23 litters) were born (Fig. 4a). 107 *Sdc1*^*+/−*^ (55%) and 101 WT (67%) mice survived and were monitored for 200 days. Statistically significant more *Sdc1*^*+/−*^ newborns died compared to WT (45% vs. 33%). The majority of pups died during the first 3 days after birth (Fig. 4b). However, the death pace between the two groups was almost the same (Fig. 4b). Reaching weaning age, 57% *Sdc1*^*+/−*^ males and 43% *Sdc1*^*+/−*^ females as well as 45% WT males and 55% WT females were separated.

**Fig. 5:**
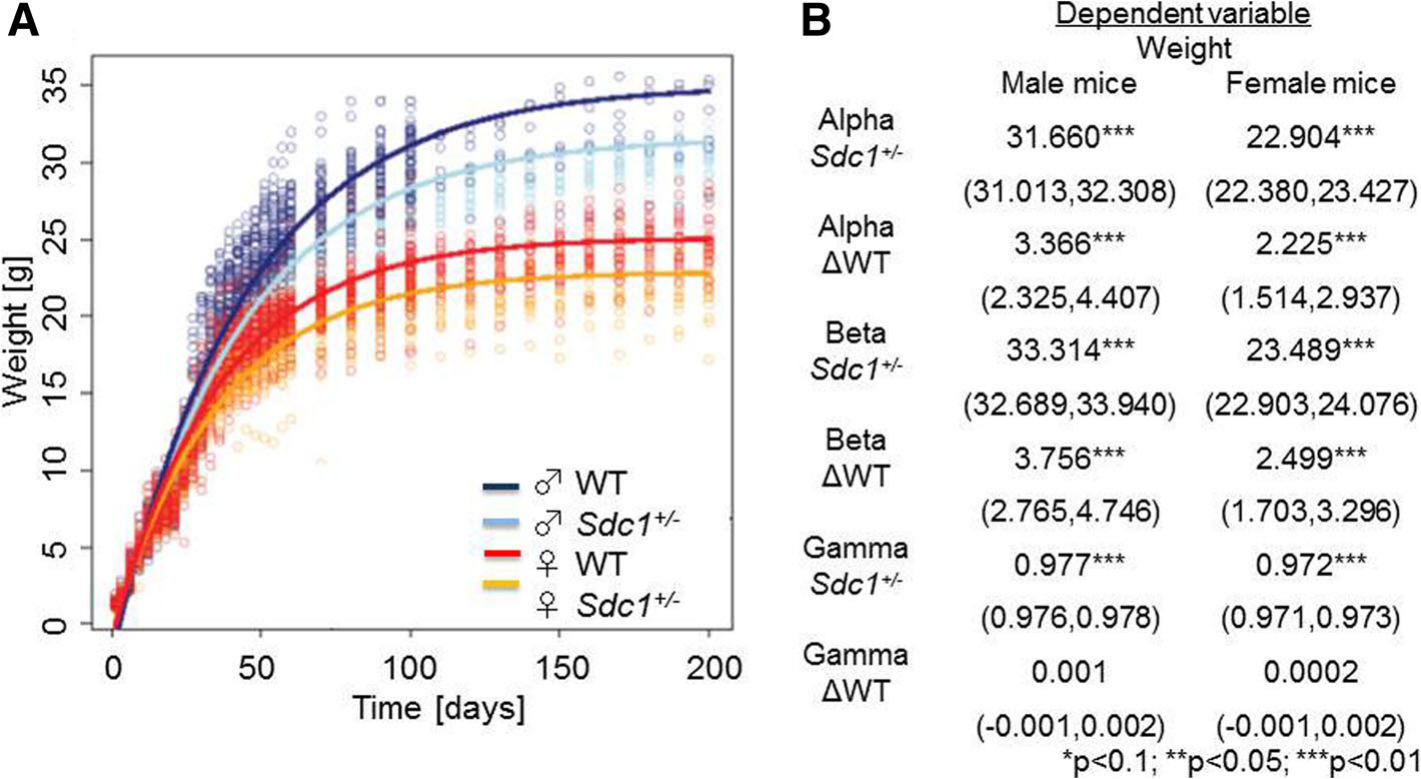
Weight curves of *Sdc1*^*+/−*^ and WT pups using a nonlinear mixed-effects model. **a** Weight is separated by gender and by mouse type as indicated with different colors observed until day 200. **b** Model for comparison of the *Sdc1*^*+/−*^ vs WT mice. The alpha (α), beta (ß) and gamma (γ) effects of the *y* = *α* − *β* ∗ *γ*^*x*^ equation for the nonlinear mixed-effects model are given with the confidence intervals and the statistical significances (**P < 0.05, ***P < 0.01)

On the day of birth, the *Sdc1*^*+/−*^ pups were significantly lighter (1.24 ± 0.01 g) than the WT pups (1.33 ± 0.01 g) (*P* < 0.001). From the day of gender determination (day 21) up to adolescence (day 200) the *Sdc1*^*+/−*^ male and female mice were 7 and 9% lighter than the WT controls respectively Single important time points during development have been selected: sexual maturity on day 33 (*Sdc1*^*+/−*^ males 17.10 ± 0.19 g and *Sdc1*^*+/−*^ females 14.58 ± 0.15 g, WT males 18.34 ± 0.38 g and WT females 15.52 ± 0.26 g) and breeding maturity on day 60 (*Sdc1*^*+/−*^ males 23.97 ± 0.15 g and *Sdc1*^*+/−*^ females 18.68 ± 0.21 g, WT males 26.00 ± 0.30 g and WT females 20.31 ± 0.23 g). At both time points, the weight difference was significantly different (*P* < 0.005). The weight gain of the mice during their development and the growth curves between the *Sdc1*^*+/−*^ and the WT control group are shown in Fig. 5a with the associated parameters (Fig. 5b). The obtained weight data displayed by the curves were also significantly different for the whole monitoring period. No significant differences in the shape and the course of the weight curves were observed. The weight of the WT mice was found in accordance with commercial breeders [32].

### Organ weight

**Fig. 6:**
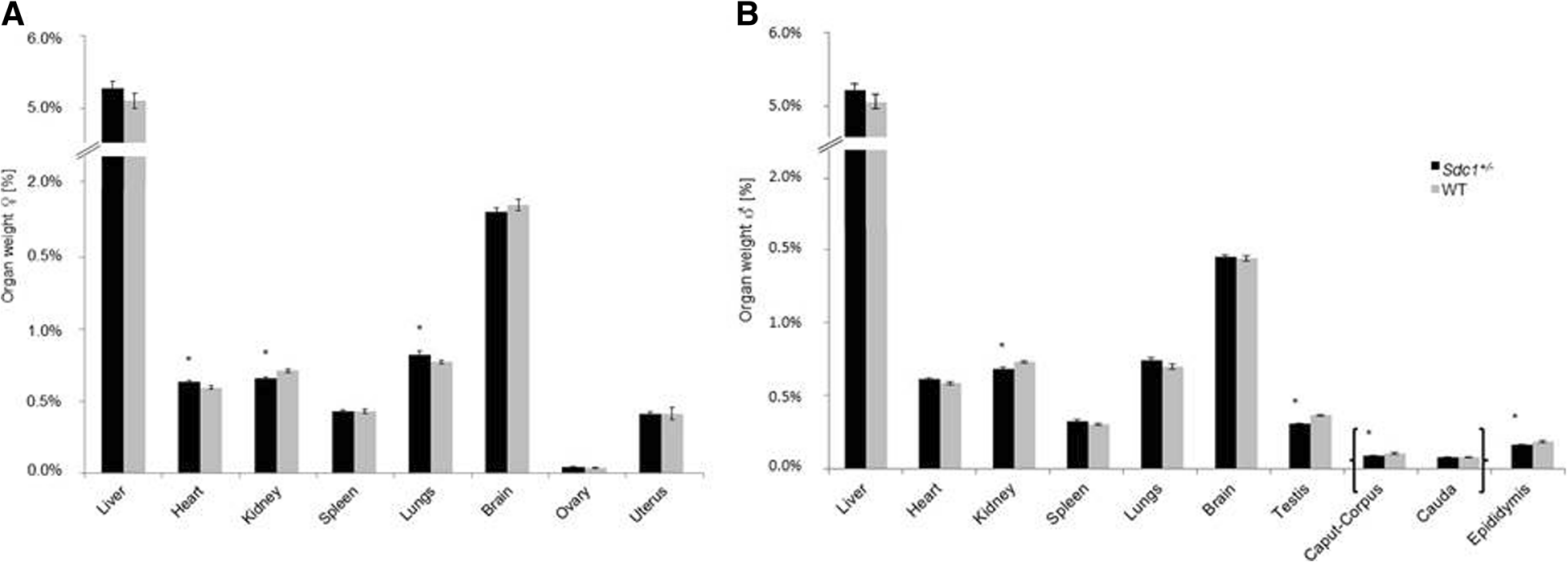
Organ per body weight for female (**a**) and male (**b**) *Sdc1*^*+/−*^ and WT mice. Organs were isolated from at least 49 *Sdc1*^*+/−*^ and WT mice. Significant organ weight differences are indicated with an asterisk (*P* < 0.05; two-tailed t-test)

Organs from at least 49 *Sdc1*^*+/−*^ and WT mice were isolated and weighed on day 200. The body weight of both *Sdc1*^*+/−*^ and WT males and females was significantly different (*P* < 0.005) (*Sdc1*^*+/−*^/WT males: 29.61 ± 0.25 g/31.61 ± 0.37 g; *Sdc1*^*+/−*^/WT females: 24.10 ± 0.27 g/25.37 ± 0.26 g). The relative values of organ weight per body weight (Fig. 6) displayed lighter kidneys and heavier hearts and lungs in the *Sdc1*^*+/−*^ females and lighter kidneys, testes and epididymis in *Sdc1*^*+/−*^ males.

### Vice versa experiment

From the vice versa embryo transfer of *Sdc1*^*+/−*^ and WT embryos a total of 19 *Sdc1*^*+/−*^ and 12 WT pups resulted, from which 26% *Sdc1*^*+/−*^and 33% WT died within the first days. Reaching weaning age, 57% *Sdc1*^*+/−*^ males and 43% *Sdc1*^*+/−*^ females as well as 38% WT males and 63% WT females were separated from their mothers.

The average duration of pregnancy for *Sdc1*^*+/−*^ foster mothers was 22.5 days (20–24 days) and for WT females 20 days (19–22 days). The *Sdc1*^*+/−*^ foster mothers gained 11.85 ± 2.34 g on average with an average number of 6 pups born. The WT foster mothers gained 13.35 ± 1.94 g on average and gave birth to an average number of 5 pups. The minimum weight gain was 9.6 g, when 6 pups were born and the maximum 17.65 g (7 pups born).

On the day of birth, the *Sdc1*^*+/−*^ pups were lighter (1.38 ± 0.04 g) than the WT pups (1.47 ± 0.05 g). In the course of growth the *Sdc1*^*+/−*^ male and female mice were 16 and 14% lighter than the WT mice respectively. On the 2 important time points, day 33 and 60, the weight differences were significantly different (day 33: *Sdc1*^*+/−*^ males 17.28 ± 1.06 g and *Sdc1*^*+/−*^ females 15.36 ± 0.53 g, WT males 22.68 ± 0.29 g and WT females 17.73 ± 0.50 g, day 60: *Sdc1*^*+/*−^ males 24.12 ± 0.31 g, *Sdc1*^*+/−*^ females 19.44 ± 0.36 g, WT males 28.47 ± 1.19 g and WT females 22.54 ± 0.38 g, *P* < 0.02).

**Fig. 7:**
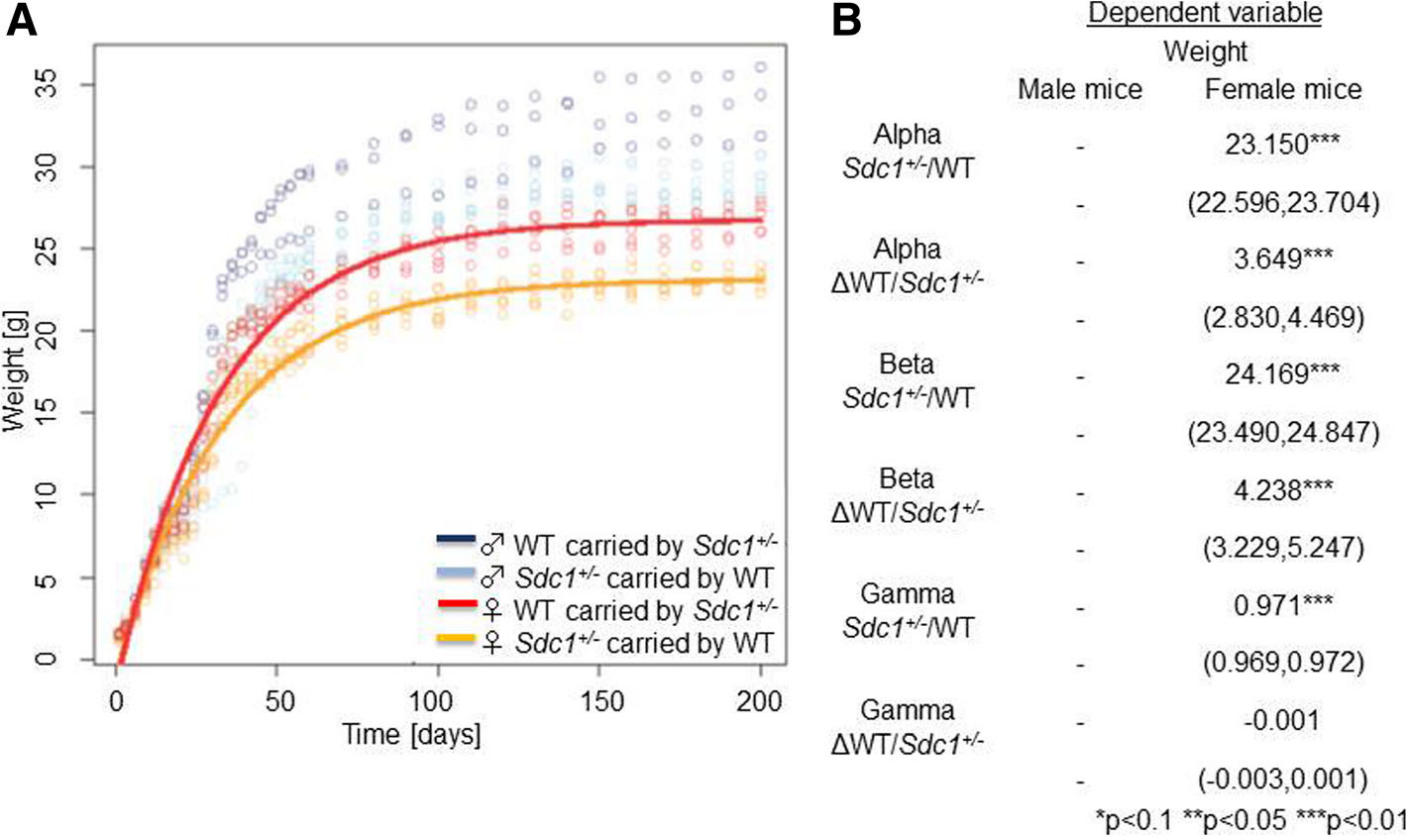
Weight curves of the *Sdc1*^*+/−*^ and WT pups after vice versa embryo transfer. **a** The weight is separated by gender and by mouse type as indicated with different colors calculated with a nonlinear mixed-effects model. **b** Comparison of the *Sdc1*^*+/−*^ vs WT mice carried by a WT or a *Sdc1*^*+/−*^ mother respectively. The alpha (α), beta (ß) and gamma (γ) effects of the *y* = *α* − *β* ∗ *γ*^*x*^ equation for the nonlinear mixed-effects model are given with the confidence intervals and the statistical significances (**P < 0.05, ***P < 0.01)

The weight gain of the vice versa mice during their development and the growth curves are displayed in Fig. 7a with significant differences (Fig. 7b). In contrast to the females, it was not possible to generate a model for the weight data of the male group, because only a few male pups were born.

**Fig. 8:**
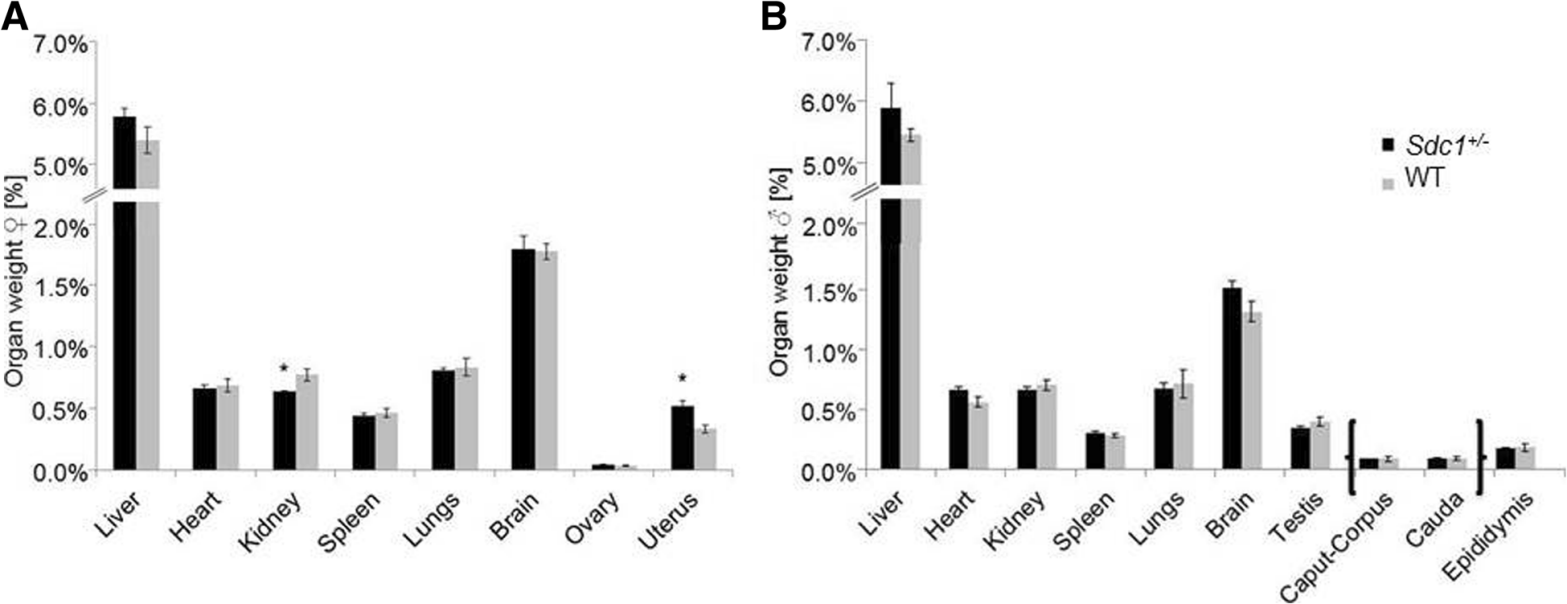
Organ per body weight after vice versa embryo transfers. Data are shown for female (**a**) and male (**b**) *Sdc1*^*+/−*^ and WT mice. Organs were isolated from all vice versa progenies (*Sdc1*^*+/−*^ males: *n* = 8, *Sdc1*^*+/−*^ females: *n* = 6; WT males: *n* = 3, WT females: *n* = 5). Significant organ weight differences are indicated with an asterisk (*P* < 0.05; two-tailed t-test)

At the age of 6 month the organs from the vice versa animals were isolated and weighed. *Sdc1*^*+/−*^ male and female mice carried by a WT mother were significantly lighter than the WT animals that were carried by a *Sdc1*^*+/−*^ mother (*Sdc1*^*+/−*^/WT males: 29.33 ± 0.36 g/34.13 ± 1.22 g; *Sdc1*^*+/−*^/WT females: 23.18 ± 0.24 g/26.98 ± 0.39 g) (*P* < 0.005). *Sdc1*^*+/−*^ females had significant lighter kidneys and significant heavier uteri (Fig. 8).

### Male reproductive characteristics

The anogenital distance showed no difference (19 vs. 20 mm). The relative weight of *Sdc1*^*+/−*^ vs. WT testis and caput-corpus per body weight was significantly different (*P* < 0.001), whereas the cauda showed no difference (Fig. 6). Histological examination of the testes also did not reveal any differences (data not shown). The sperm concentration of motile and non-motile spermatozoa did not differ, however a higher percentage of motile spermatozoa existed in the *Sdc1*^*+/−*^ males. The percentage of vital and dead sperm also did not differ (Table 2).

**Table 2:** Concentration of motile, non-motile, vital und non-vital spermatozoa from *Sdc1*^*+/−*^ and WT males

	Motile Mio/ml (%)	Non-motile Mio/ml (%)	Vital Mio/ml (%)	Non-vital Mio/ml (%)
*Sdc1* ^*+/−*^	1.49 ± 0.09 (43.95)	2.10 ± 0.19 (56.05)	6.07 ± 0.19 (87.08)	0.89 ± 0.06 (12.92)
WT	1.69 ± 0.17 (41.04)	2.33 ± 0.18 (58.96)	5.91 ± 0.23 (88.59)	0.78 ± 0.06 (11.41)

**Fig. 9:**
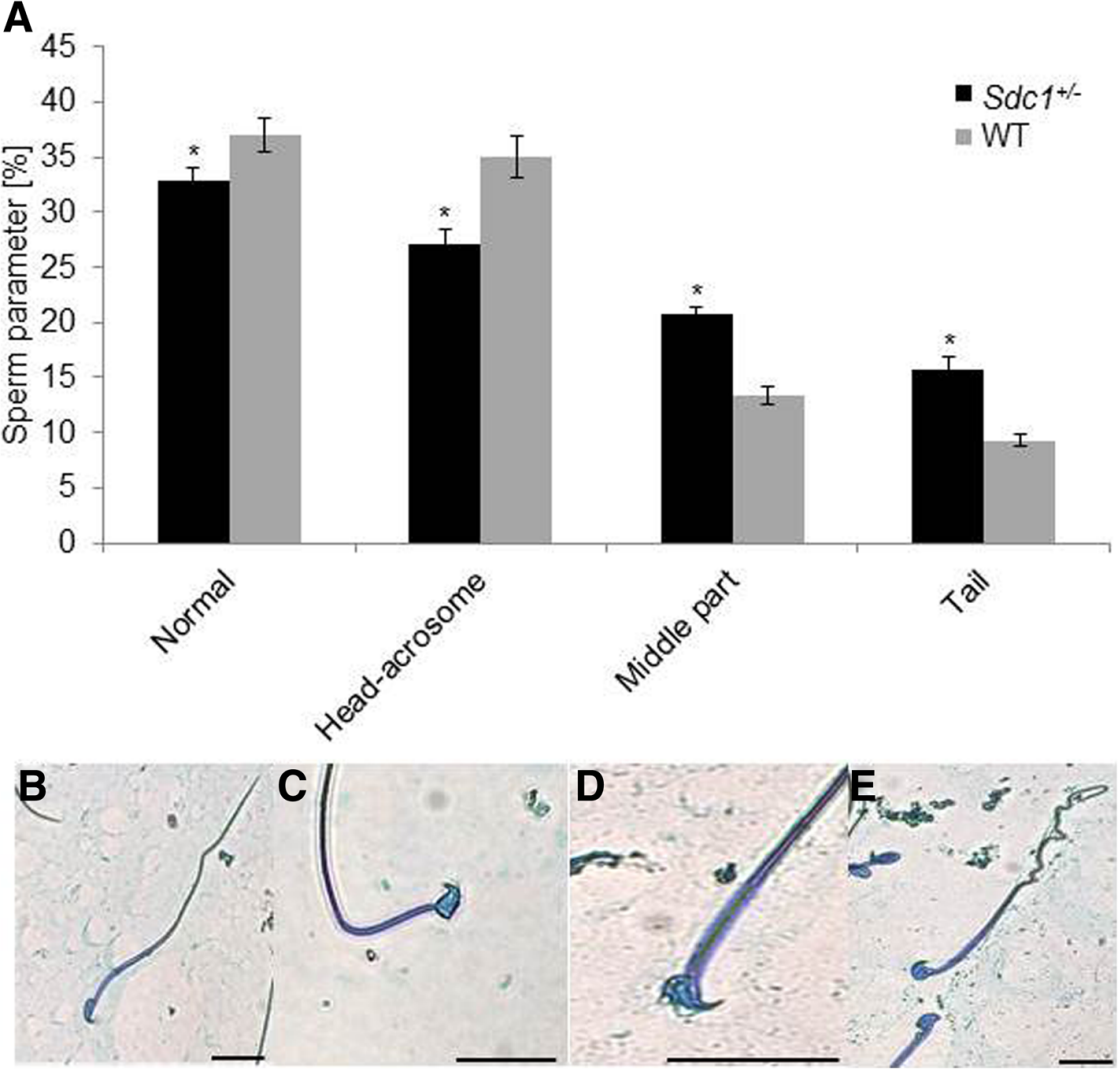
Comparison of the sperm abnormalities observed between *Sdc1*^*+/−*^ and WT males. **a** Spermatozoa with head-acrosome-, midpiece- and tail-deficiencies were observed opposed to the normal ones. Spermatozoa with more than one defect were not assigned to one of these categories (*Sdc1*^*+/−*^/WT males: *n* = 28/24; all mice sexually matured: 6–12 months; technical repeats: *n* = 2; number of spermatozoa counted: *n* = 100; *P* < 0.05; chi square test). **b**-**e** Representative photos of the observed abnormalities (bar = 8 μm, normal spermatozoon (**b**), spermatozoon with head (**c**), acrosome (**d**) or tail (**e**) defect)

Concerning the morphology, the spermatozoa of the *Sdc1*^*+/−*^ males demonstrated a higher number of abnormalities compared to WT. The *Sdc1*^*+/−*^ spermatozoa had more midpiece and tail abnormalities, whereas the WT spermatozoa showed more head-acrosome deficiencies (Fig. 9a, b).

## Discussion

The importance of the SDC1 protein and its involvement in human pregnancy associated pathologies in human elucidates the necessity of using a suitable animal model. The individual analysis of the different maternal and paternal reproductive characteristics was performed in *Sdc1*^*+/−*^ mice to enlighten the reproductive phenotype taking into account that a complete loss of SDC1 seems to be unlikely in human.

Selected findings are discussed further in the following paragraphs:

### Mouse cycle

An easy-to-interpret marker in mouse breeding is the vaginal estrous cycle, which can be predicted through changes in the morphology and content of vaginal cells [33]. The objective of the estrous cycle monitoring was to determine the influence of the reduced expression of SDC1 on cycle frequency and length, as studies on selected lines examined for fecundity revealed a correlation between cyclicity and reproductive performance [34].

The estrous cycle for *Sdc1*^*+/−*^ and WT mice lasted 5 days on average, which is in accordance with data from the literature [35] and the Mouse Genome Informatics Jackson Laboratory Database [36]. Interestingly, the WT females went through more complete regular cycles compared to *Sdc1*^*+/−*^. Correspondingly, a significant higher percentage of the *Sdc1*^*+/−*^ females showed a 6 day long estrous cycle. Among the *Sdc1*^*+/−*^ females, a significantly prolonged P stage was observed suggesting a delayed ovulation or a longer maturation of the ovarian follicles. During the E stage, the females are more receptive to males and copulation is more likely to happen. Although *Sdc1*^*+/−*^ females showed less E stages, no impact on the pregnancy rate occurred.

### Characteristics of the female reproductive phenotype and progeny

A former study on the role of the heparin-binding EGF-like growth factor showed that the HSPG may be beneficial for blastocyst endometrial interaction in mice [37]. The average duration of a pregnancy for both *Sdc1*^*+/−*^ and WT females was in accordance to the Jackson Laboratory database (18 to 22 days).

Concerning litter sizes and therefore an indirect indicator for breeding quality, the sizes were in accordance to the MGI international database resource [38], but a significantly higher number of *Sdc1*^*+/−*^ pups died postnatally within the first 7 days. A litter loss of 32% for C57BL/6 mice described in the literature is in accordance to our data for the WT animals [39]. Mammal pups depend on their mother for nutrition and the absence of lactation could lead to death [40]. Although the mammary glands of the *Sdc1*^*−/−*^ females are hypomorphic [3], our vice versa experiment showed, that still 26% of the *Sdc1*^*+/−*^ pups died when carried and nursed by a WT foster mother which rather hints to a genotype-association rather than a lactation problem. The lower number of postnatally dead pups from the vice versa setting led to the hypothesis that there might be an additive maternal effect though. Hence it is of great interest that former studies on *Sdc1*^*−/−*^ mice revealed that these mice show symptoms of abnormal cold stress at normal housing temperatures and have an impaired intradermal adipocyte function [41]. These findings and the already proven importance of the brown adipocyte tissue for the survival of newborn pups [42] might rather explain the increased death rate of the *Sdc1*^*+/−*^ pups.

In our study, the *Sdc1*^*+/−*^ mice were systematically smaller, either when carried by a *Sdc1*^*+/−*^ or a WT foster mother. In contrast to the females, it was not possible to generate a model for the weight data of the male group, because only a few male pups were born. It is worth mentioning here that both *Sdc1*^*+/−*^ and WT mice showed a similar course of weight gain during the 200 days which is congruent to the literature for the WT mice [43].

### Organ weight

The relative values of organ to body weight were calculated to erase a possible bias concerning the lighter body weight of the *Sdc1*^*+/−*^. The relative kidney weight of *Sdc1*^*+/−*^ mice as well as *Sdc1*^*+/−*^ females resulting from vice versa transfers was significantly lower than in the WT animals. Reduced SDC1 expression could influence epithelial-mesenchymal interaction being important for kidney morphogenesis [44] with a possible widespread impact on the kidney physiology since the kidney has been found to be a source considerably rich in SDC1 [45]. Possible alterations in the HS structure, as in the case of the 2-O-sulfotransferase-deficient embryos, may influence the binding of growth factors and morphogens that are important for kidney development [46]. Previous studies have shown an impaired renal function associated with a reduced tubular repair [47] possibly similar to SDC1’s role in dermal wound healing [20].

The mouse testes weight is directly correlated to male fertility, i.e., spermatogenic ability [48]. SDC1 could be associated to rat sertoli cell development [49] and maturation being a target and co-receptor of bFGF [50] suggesting a potential role for SDCs in spermatogenesis. The size and weight of the testes of WT males were in accordance to other mouse strains [51]. Intriguingly, the *Sdc1*^*+/−*^ relative testis weight was significantly lower although the anogenital distance as a marker for male masculinization programming window during embryogenesis [52] showed no difference. The reproductive outcome observed by implantation sites and litter sizes of the *Sdc1*^*+/−*^ mice was not impaired.

## Conclusions

In conclusion, we demonstrated that the reduced expression of SDC1 impairs the reproductive phenotype resulting in more postnatally dead pups and a genotype-related reduced body weight including some organs throughout the lifespan of the mice. Further studies need to elucidate the origin of the observations and therefore gaining more insight into the role of SDC1 in the hormonal axis, signaling pathways and cellular effects.

## Abbreviations

D: Diestrus
E: Estrous
EGF: Epidermal growth factor
HCG: human chorionic gonadotrophin
HELLP: Hemolysis, elevated liver enzymes and low platelet count
HS: Heparan sulfate
KO: Knock-out
M: Metestrus
P: Proestrus
PG: Proteoglycan
PMSG: Pregnant mare’s serum gonadotrophin
Sdc1: Syndecan-1
WT: Wild type
